# CUDC‐907 blocks multiple pro‐survival signals and abrogates microenvironment protection in CLL

**DOI:** 10.1111/jcmm.13935

**Published:** 2018-10-24

**Authors:** Yixiang Chen, Chloé Peubez, Victoria Smith, Shiqiu Xiong, Gabriella Kocsis‐Fodor, Ben Kennedy, Simon Wagner, Constantine Balotis, Sandrine Jayne, Martin J. S. Dyer, Salvador Macip

**Affiliations:** ^1^ Mechanisms of Cancer and Ageing Laboratory Department of Molecular and Cell Biology University of Leicester Leicester UK; ^2^ Ernest and Helen Scott Haematological Research Institute University of Leicester Leicester UK; ^3^ Medical College Henan University of Science and Technology Luoyang China; ^4^ Department of Cancer Studies University of Leicester Leicester UK; ^5^ Department of Haematology University Hospitals of Leicester Leicester UK

**Keywords:** CLL, HDAC, microenvironment, PI3K

## Abstract

CUDC‐907, a dual PI3K/HDAC inhibitor, has been proposed to have therapeutic potential in hematopoietic malignancies. However, the molecular mechanisms of its effects in chronic lymphocytic leukaemia (CLL) remain elusive. We show that CLL cells are sensitive to CUDC‐907, even under conditions similar to the protective microenvironment of proliferation centres. CUDC‐907 inhibited PI3K/AKT and HDAC activity, as expected, but also suppressed RAF/MEK/ERK and STAT3 signalling and reduced the expression of anti‐apoptotic BCL‐2 family proteins BCL‐2, BCL‐xL, and MCL‐1. Moreover, CUDC‐907 downregulated cytokines BAFF and APRIL and their receptors BAFFR, TACI, and BCMA, thus blocking BAFF‐induced NF‐κB signalling. T cell chemokines CCL3/4/17/22 and phosphorylation of CXCR4 were also reduced by CUDC‐907. These data indicated that CUDC‐907 abrogates different protective signals and suggested that it might sensitize CLL cells to other drugs. Indeed, combinations of low concentrations of CUDC‐907 with inhibitors of BCL2, BTK, or the NF‐κB pathway showed a potent synergistic effect. Our data indicate that, apart from its known functions, CUDC‐907 blocks multiple pro‐survival pathways to overcome microenvironment protection in CLL cells. This provides a rationale to evaluate the clinical relevance of CUDC‐907 in combination therapies with other targeted inhibitors.

## 1 INTRODUCTION

Chronic lymphocytic leukaemia (CLL) is characterized by a progressive accumulation of monoclonal B cells, the survival and proliferation of which are highly dependent on interactions with the microenvironment.^1^, ^2^ CLL cells in bone marrow and lymphoid node proliferation centres display higher chemoresistance and proliferative capacity than cells in the peripheral blood.^3^ In these protective microenvironments, CLL cells interact with several accessory cells, such as mesenchymal marrow stromal cells, follicular dendritic cells, monocyte‐derived nurse‐like cells (NLCs), and T cells, and receive from them a diverse range of protective, anti‐apoptotic signals.^3^, ^4^, ^5^, ^6^, ^7^

Various cytokines, chemokines, and adhesion molecules are involved in these microenvironment interactions, including tumour necrosis factor (TNF) family members BAFF (B‐cell activating factor of the TNF family) and APRIL (a proliferation‐inducing ligand).^8^, ^9^ The secretion of T cell chemokine (C‐C motif) ligand 3 (CCL3) and CCL4/22 by CLL cells is also important for adhesion, retention, and tissue homing.^10^ Moreover, in this protective niche, bone marrow stromal cells, and NLCs constitutively secrete the chemokines CXCL12 (also known as stroma‐cell derived factor, SDF‐1) and CXCL13.^11^ These activate the surface membrane receptor CXCR4 (sCXCR4) in CLL cells, inducing chemotaxis and migration.^12^, ^13^

Multiple signalling pathways, including PI3K/AKT, RAF/MEK/ERK, STAT3, and NF‐κB, are constitutively activated in CLL cells.^14^, ^15^, ^16^, ^17^, ^18^, ^19^ Given the pivotal role of PI3K/AKT in cancer initiation, growth, proliferation, and survival, an increasing number of small molecular inhibitors that target this pathway have been developed. For example, a PI3Kδ specific inhibitor (CAL‐101/idelalisib) has shown promising results in the treatment of patients with relapsed/refractory CLL.^20^, ^21^ However, the efficacy of PI3K inhibitors, like most targeted drugs, is limited by concurrent activation of other pro‐survival and growth‐related pathways that lead to drug resistance.^22^ A potential strategy to overcome these limitations is combining PI3K inhibition with drugs that target other pathways, in order to achieve synergistic anti‐tumour activity.

HDAC inhibitors can regulate both histone and non‐histone protein substrates through epigenetic or non‐epigenetic modification, thus affecting multiple signalling networks.^23^, ^24^ Several pre‐clinical studies of HDAC inhibitors, alone or in combination with other anti‐cancer drugs, have shown promising results for the treatment of hematologic malignancies.^25^ For example, simultaneous inhibition of HDAC and PI3K/mTOR signalling with panobinostat and rapamycin has been shown to synergistically inhibit tumour cell growth and induce apoptosis in diffuse large B cell lymphoma (DLBCL) 26 as well as in solid tumour cells.^27^

CUDC‐907 is a dual inhibitor targeting class I PI3Ks as well as class I and II HDACs, and has been reported to induce apoptosis in various cancer cells in vitro.^28^ Recent clinical trials showed the safety, tolerability, and a promising activity of CUDC‐907 in patients with relapsed or refractory lymphoma or multiple myeloma,^29^ or DLBCL, particularly in MYC mutant malignancies.^30^ In this study, we investigated the efficacy of CUDC‐907 in CLL cells and found that it effectively disrupted multiple pro‐survival signalling pathways to overcome the microenvironment protection and induce apoptosis. This suggest a new mechanism of action of CUDC‐907 in the context of B cell malignancies and indicates the need for further assessment of this drug in patients, especially in combination with other targeted therapies.

## 2 METHODS

### 2.1 Patient samples

Peripheral blood samples were obtained from CLL patients attending clinics at the Leicester Royal Infirmary (Leicester, UK) following informed consent and approval from the local Research Ethics Committee and in accordance with the Declaration of Helsinki. All patients, diagnosed according to IWCLL‐NCI 2008 guidelines,^31^ were treatment free for at least 6 months and had a cell count >50 × 10^9^/L. Peripheral blood mononuclear cells were separated from whole blood by density centrifugation. Heparinized whole blood was diluted 1:1 with PBS and gently layered onto 15 mL Ficoll (Histopaque 1077; Sigma‐Aldrich, Poole, UK) prior to centrifugation at 400 *g* for 30 minutes. The mononuclear cell layer was removed from the interphase, washed and resuspended in RPMI‐1640 medium (Life Technologies, Paisley, UK) supplemented with 10% fetal bovine serum (Lonza, Slough, UK), L‐Glutamax (2 mmol/L), penicillin (50 U/mL) and streptomycin (50 mg/mL). The isolated mononuclear cells had a CLL cell purity of >90% in all cases, as determined by flow cytometry.

### 2.2 Cells, reagents and inhibitors

Chronic lymphocytic leukaemia cells were cultured in RPMI‐1640 medium containing soluble 10 ng/mL interleukin (IL)‐4 and CD40 ligand (CD40L or CD154) to mimic the microenvironment of proliferation centres^3^, ^4^ as previously described.^17^ Cells were incubated for 24 hours in these conditions before applying any treatments. Human CLL cell line MEC‐1 was cultured as previous described.^17^ Goat F(ab’)2 anti‐human IgM was purchased from Bio‐Rad (Hercules, CA, USA), recombinant human BAFF (soluble) was purchased from Enzo (Farmingdale, NY, USA). CUDC‐907, IMD‐0354, ABT‐199, Ibrutinib, Entospletinib, CAL‐101/idelalisib, and PLX‐4720 were obtained from Selleckchem (Houston, TX, USA). HCT116 colon cancer cells were cultured in DMEM medium containing 10% of FCS and penicillin/streptomycin (50 U/mL).

### 2.3 Assessment of cell viability and death

Cell viability was assessed by the CellTiter 96 Aqueous One Solution Cell Proliferation MTS Assay (Promega, Madison, WI, USA), following the manufacturer's instructions as previously described.^17^ The absorbance at 490 nm was recorded on a TECAN infinite F50 reader (Labtech International, Heathfield, UK). These experiments were performed in triplicate and repeated on at least two independent occasions. Cell death was measured by staining with propidium iodide (PI) for 30 minutes at 4°C. The percentage of PI‐positive cells (dead) determined by flow cytometry using a FACS Canto II cytometer (BD Biosciences, Franklin Lakes, NJ, USA). Alternatively, apoptosis was measured by Annexin V staining, as previously described.^17^

### 2.4 Western blotting

Total protein was extracted from cells lysates using RIPA lysis buffer and loading buffer as previous described.^17^ Proteins were separated with SDS‐PAGE and incubated with specific antibodies. Protein bands were visualized and quantified with an Odyssey system (Pierce, Waltham, MA, USA). The antibodies used were: AKT, phospho‐AKT (Ser473), phospho‐p70S6K1 (Thr389), ERK, phospho‐ERK (Thr202/Tyr204), MEK1/2, phospho‐MEK1/2 (Ser271/221), IκBα, phospho‐IκBα (Ser32/36), STAT3, phospho‐STAT3 (Tyr705), caspase 9, caspase 8, and PARP, all obtained from Cell Signaling Technology (Danvers, MA, USA). BCL‐xL/S, MCL‐1, NF‐κB(p65), NF‐κB(RelB), and CXCR4(4G10) were bought from Santa Cruz Biotechnology (Dallas, TX, USA); Ac‐H3K9 obtained from Active Motif (Carlsbad, CA, USA); Phospho‐CXCR4 (S339) (ab74012) was purchased from Abcam (Cambridge, UK); β‐actin was obtained from Millipore (Burlington, MA, USA); The BCL‐2 antibody was purchased from Dako (Agilent Technologies, Santa Clara, CA, USA). Fluorescent‐conjugated secondary anti‐rabbit or anti‐mouse antibodies were purchased from Enzo life sciences.

### 2.5 Chemokine secretion

6 × 10^5^ CLL patient cells were cultured in 96‐well plates. Cells were stimulated with anti‐IgM (10 μg/mL) and various concentration of CUDC‐907 for 24 hours, then the supernatant were collected and the secretion of CCL3/4 was measured by quantitative ELISA. ELISA–related products, including human CCL3/MIP‐1α DuoSet ELISA, human CCL4/MIP‐1 beta DuoSet ELISA, and DuoSet ancillary reagent Kit 2, were purchased from R&D Systems. The plate preparation and assay protocol were conducted according to the manufacturer's instructions (R&D Systems, Minneapolis, MN, USA).

### 2.6 Surface membrane CXCR4 expression

3 × 10^6^ CLL patient cells were cultured in a 24‐well plate. Cells were either stimulated with 200 ng/mL SDF‐1α (CXCL12) (Upstate Biotechnology, Thermo Fisher Scientific, Waltham, MA, USA) or received no stimulation. Simultaneously, cells were treated with CUDC‐907 (concentrations ranging from 0.001 to 1 μmol/L) or DMSO (control) for 12 hours. Then, cells were collected, washed, and resuspended in cold PBS. A CXCR4 primary antibody (Santa Cruz Biotechnology) was added (5‐10 μg/mL). After incubated on ice for 30 minutes, cells were washed with cold PBS and incubated with fluorescent labelled secondary antibody (10 μg/mL) on ice for 30 minutes in the dark. The expression of sCXCR4 was measured by flow cytometry with a FACS Canto II cytometer (BD Biosciences).

### 2.7 Quantitative real‐time PCR

Total RNA was extracted using the ReliaPrepTM RNA Cell Miniprep System (Promega). cDNA was obtained using Thermo Script reverse transcriptase (SuperScript III First‐Strand Synthesis System; Invitrogen, Carlsbad, CA, USA). Quantitative real‐time PCR (qPCR) was performed on a Roche LightCycler using the sensiMix SYBR No‐Rox kit following the manufacturer's protocol (Roche, Basel, Switzerland). The primers sets used are shown in Table S1. Relative gene expression was calculated on the basis of the threshold cycle (C_t_) values and normalization of internal control expression using the 2^−ΔΔCt^ method.^32^ The internal control housekeeping gene used in this study was glyceraldehyde‐3‐phosphate dehydrogenase (GAPDH). Experiments were performed in triplicate and repeated three times.

## 3 RESULTS

### 3.1 CUDC‐907 induces CLL cell death

**Figure 1:**
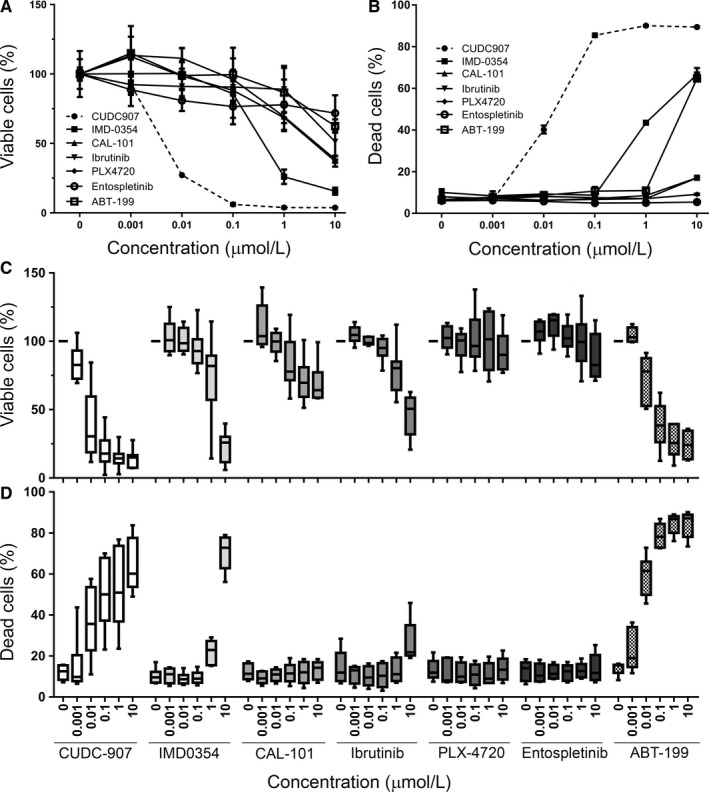
CUDC‐907 inhibits CLL cell growth and induces apoptosis. A, MEC‐1 cell viability measured by MTS assay, normalized to Control samples (0 μmol/L). Cells were treated with various small molecule inhibitors (0.001‐10 μmol/L) or DMSO (0 μmol/L) for 48 h. Experiments were performed in triplicate and repeated at least 2 times. Graphics show mean values and error bars represent standard deviation, as in all the other panels of this figure. B, Induction of cell death in MEC‐1, as measured by FACS analysis of by PI‐stained cells. Cells were treated with inhibitors for 48 h. Graphics show percentage of PI positive (dead) cells. C, Same as (A), using primary CLL cells. Cells were cultured for 24 h in the presence of 10 ng/mL IL‐4 and CD154 and then treated with inhibitors for 48 h. The number of primary samples used for each inhibitor is: 23 (CUDC‐907), 13 (IMD0354), 6 (CAL‐101), 11 (Ibrutinib), 8 (PLX‐4720), 6 (Entospletinib), and 7 (ABT‐199). Experiments were performed in triplicate. Graphics show mean values and error bars represent standard deviation. D, Same as (B), using primary CLL cells. The number of primary samples used for each inhibitor are: 8 (CUDC‐907), 6 (IMD0354), 5 (CAL‐101), 5 (Ibrutinib), 5 (PLX‐4720), 5 (Entospletinib), and 5 (ABT‐199)

To uncover potential new therapies for B cell malignancies, we screened the MEC‐1 CLL cell line 33 for its response to different clinically relevant targeted inhibitors, including entospletinib (SYK inhibitor), ibrutinib (BTK inhibitor),^34^, ^35^ PLX‐4720 (BRAF specific inhibitor), CAL‐101/Idelalisib (PI3Kδ inhibitor),^20^, ^21^ ABT‐199 (BCL2 inhibitor),^36^ IMD‐0354 (NF‐κB inhibitor),^37^ and CUDC‐907 (PI3K/HDAC inhibitor).^28^ As shown in Figure 1A and B, CUDC‐907 was more effective than all the other inhibitors tested at inducing cell death in the MEC‐1 cell line, with an EC50 at nanomolar concentrations. We confirmed these results in CLL patient cells cultured with CD154 and IL‐4, a combination of factors that mimics some of the main features of the proliferation centre microenvironment.^38^, ^39^ In these conditions, CUDC‐907 was shown to be one of the most efficient at reducing cell viability and induce cell death as well, which also happened at nanomolar concentrations and in a dose‐dependent manner (Figure 1C and D). Of note, the sensitivity of CLL cells to BCL‐2 inhibitors shown in these figures is consistent with previous reports.^40^ These data suggest that CLL cells are highly sensitive to CUDC‐907 at concentrations that are within the expected therapeutic levels that can be achieved in vivo, which range from 27 to 70 ng/mL.^29^, ^30^

### 3.2 CUDC‐907‐induced CLL cell death is characterized by the inhibition of multiple pro‐survival signals

**Figure 2:**
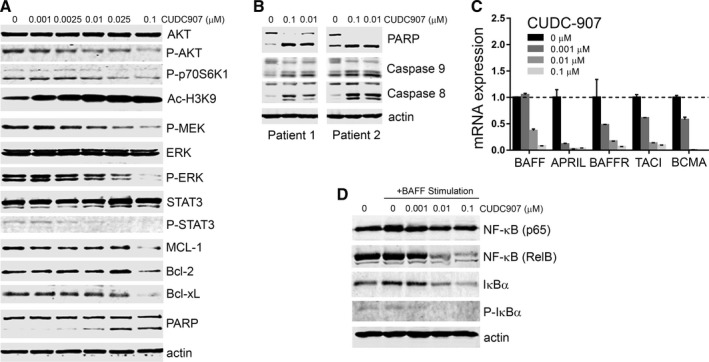
CUDC‐907 inhibits pro‐survival signals in CLL cells. A, Western blots showing protein expression in lysates of MEC‐1 cells treated with CUDC‐907 at various concentrations or DMSO as a control (0) for 12 h. AKT, p‐AKT (Ser473), p‐p70S6K (Thr389), Ac‐H3K9, ERK, p‐ERK (Thr202/Tyr204), p‐MEK (Ser217/221), STAT3, p‐STAT3 (Tyr705), MCL‐1, BCL‐2, BCL‐xL, and PARP were detected with specific antibodies. β‐actin was used as a loading control. B, Representative Western blot showing PARP, caspase‐9, and caspase‐8 cleavage in CLL primary cells from two patients. Cells were cultured as in Figure 1C and treated with different concentrations of CUDC‐907 for 12 h. C, mRNA expression levels of BAFF, APRIL, BAFFR, TCIA, and BCMA, as measured by qRT‐PCR, in CLL patient cells cultured in the presence of 10 ng/mL IL‐4 and CD154 for 24 h then treated with CUDC‐907 for 12 h. The expression of target genes was normalized to an internal control, GAPDH. Experiments were performed in triplicates and repeated at least 3 times. Data were expressed as relative to control (no inhibitor). D, Representative Western blot of lysates of CLL patient cells stimulated with BAFF (100 ng/mL) for 24 h. NF‐κB(p65), NF‐κB(RelB), IκBα, p‐IκBα, and β‐actin were detected using specific antibodies. The graph shows quantitation the Western blot bands normalized to β‐actin and expressed as relative to control (0 h) treatment

We next investigated the mechanisms that could be involved in the effects of CUDC‐907 on CLL cell viability. We first tested whether it had any impact on the activation of signalling pathways essential for CLL cell proliferation and survival. As shown in Figure 2A and Figure S1, CUDC‐907 decreased the phosphorylation of AKT (Ser473) and p70S6K1 (Thr389), the downstream effectors of the PI3K cascade, and greatly increased the acetylation of H3K9 (Ac‐H3K9). This confirms the inhibition of the PI3K and HDAC activities by CUDC‐907 and is consistent with previous observations in other cancer cells.^28^ In addition, we also found that CUDC‐907 decreased ERK (Thr202/Tyr204), MEK1/2 (Ser271/221), and STAT3 (Tyr705) phosphorylation, suggesting a blockage of both the RAF/MEK/ERK and STAT3 signalling pathways. The accumulation of the anti‐apoptotic BCL‐2 family proteins, such as BCL2, BCL‐xL, and MCL‐1, plays a role in the acquirement of resistance to spontaneous and drug‐induced apoptosis in CLL.^41^, ^42^, ^43^ We explored the effects of CUDC‐907 on these anti‐apoptotic proteins and found that it only affected their expression at the higher concentrations tested (Figure 2A and Figure S1), which suggests that it may not be an essential requirement for the induction of cell death here. Of note, CUDC‐907 induced PARP cleavage, indicating that cell death was likely caused by the activation of the apoptotic cascade. Consistent with this, we also found that CUDC‐907 induced cleavage of caspase 8 and caspase 9 and PARP in CLL cells, which suggests the implication of both the intrinsic and extrinsic apoptotic pathways (Figure 2B). In order to further explore this finding, a colon cancer cell line that lacks Bax (HCT116 Bax^−/−^)^44^ was treated with CUDC‐907. It showed significant resistance to cell death, as compared to an isogenic wild type line (Figure S2), indicating that CUDC‐907‐induced apoptosis is preferentially but not exclusively mediated by the Bax‐dependent pathway. This is consistent with the implication of both the intrinsic and extrinsic pathways in the induction of cell death by CUDC‐907. Together, these results show that exposure to CUDC‐907 leads to the inhibition of different pro‐survival signalling pathways in CLL cells, which likely contributes to an apoptotic response carried mostly through the intrinsic pathway, but with involvement of the extrinsic pathway as well.

### 3.3 CUDC‐907 impairs NF‐κB signalling by decreasing the expression of BAFF, APRIL, and their receptors

To determine whether CUDC‐907 also has an effect on the interaction of CLL cells with the microenvironment, which is an important source of protective stimuli, we measured the expression of cytokines BAFF and APRIL and their receptors BAFF‐R (B cell‐activating factor receptor), TACI (transmembrane activator of the calcium modulator and cyclophilin ligand‐interactor), and BCMA (B cell mutation antigen).^8^, ^9^ These proteins belong to the TNF superfamily and are important for canonical NF‐κB pathway activation.^45^ As shown in Figure 2C and Figure S3A, CUDC‐907 significantly decreased the expression of all these proteins. We next evaluated the effects of CUDC‐907 after BAFF stimulation, in order to assess its impact on the level of NF‐κB signalling. As expected, BAFF ligand stimulation induced NF‐κB (p65), IκBα, and phospho‐IκBα expression, suggesting NF‐κB activation (Figure 2D and Figure S3B). However, CUDC‐907 decreased the expression of NF‐κB (p65), NF‐κB (RelB), IκBα, and phospho‐IκBα in a dose‐dependent manner. These results together suggest that CUDC‐907 inhibits the NF‐κB signalling pathway, which may reduce the protection of CLL cells in proliferation centres and thus contribute to the induction of apoptosis.

### 3.4 CUDC‐907 modulates the expression of secreted microenvironmental factors

**Figure 3:**
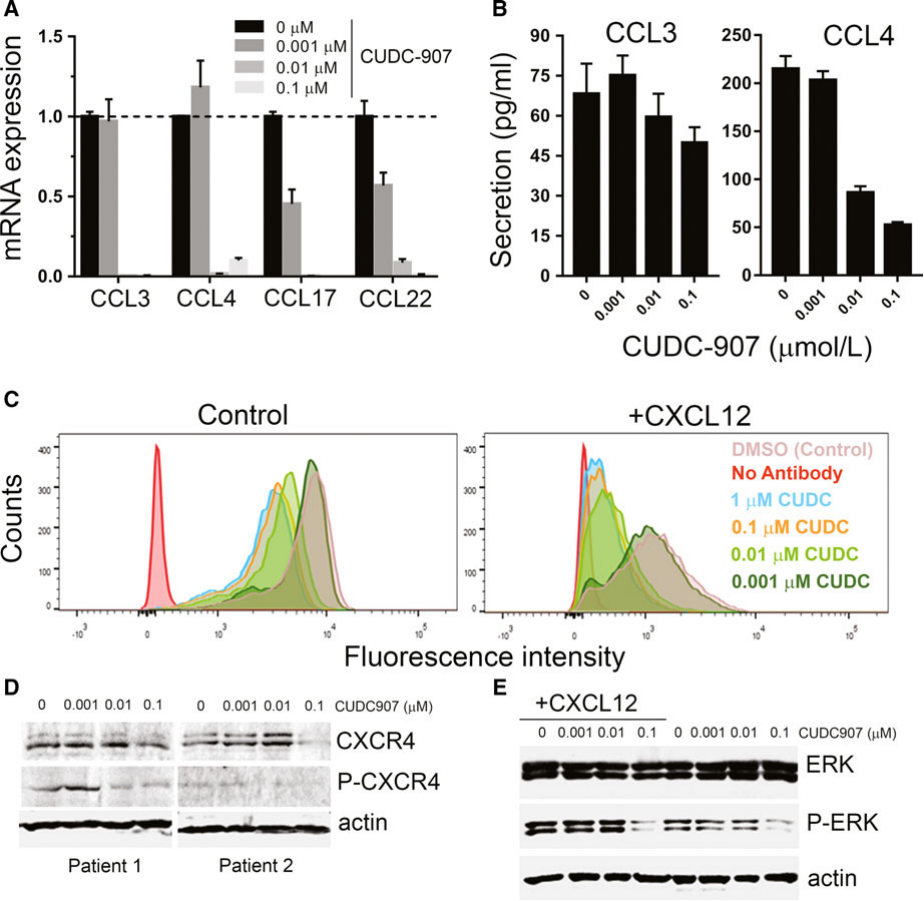
Effects of CUDC‐907 on the expression of microenvironment chemokines. A, mRNA expression of CCL3, CCL4, CCL17, and CCL22, as measured by qRT‐PCR in CLL patient cells cultured with 10 μg/mL anti‐IgM and treated with CUDC‐907 for 24 h. B, Secretion levels of CCL3/4, as measured by quantitative ELISA, in supernatants of CLL patient cells cultured with anti‐IgM and treated with CUDC‐907. C, Expression of smCXCR4 in CLL patient cells not stimulated (left) or stimulated with 200 ng/mL CXCL12 (right), and treated with CUDC‐907 for 12 h. DMSO was used in controls. Cells were incubated with a CXCR4 primary antibody and a fluorescent labelled secondary antibody. The smCXCR4 signalling was measured with FACS. D, Representative Western blot showing levels of total and phosphorylated CXCR4 measured in lysates of CLL cells from two patient treated with CUDC‐907 for 12 h. E, Representative Western blot showing levels of ERK and phospho‐ERK in CLL patient treated as in (C)

Chronic lymphocytic leukaemia cells express high levels of BCR signalling‐activated T‐cell chemokines (such as CCL3, 4, 17, and 22), which are critical for creating a protective microenvironment.^46^, ^47^, ^48^ We, therefore, examined the effect of CUDC‐907 on these chemokines in response to BCR engagement (with anti‐IgM stimulation, which has been reported to induce CCL3/4 expression^46^). As shown in Figure 3A, CUDC‐907 decreased CCL3/4/17/22 expression. Moreover, CUDC‐907 inhibited CCL3/4 secretion in a dose‐dependent manner (Figure 3B).

The CXCR4‐CXCL12 axis is critical for the interaction between CLL cells and stromal cells.^10^, ^11^, ^12^ We found that CUDC‐907 reduced the expression of sCXCR4 (Figure 3C). This was also evident after stimulation with CXCL12, a ligand of CXCR4 that promotes sCXCR4 receptor internalization.^12^ In CLL patient cells, the overexpression of sCXCR4 correlates with hyper‐phosphorylated Ser339 residues.^49^ We observed that CUDC‐907 importantly decreased the phosphorylation level of CXCR4 at Ser339 (Figure 3D). Total CXCR4 levels were also reduced by CUDC‐907 at higher concentrations. Since the binding of CXCL12 to CXCR4 activates different downstream signalling pathways, including RAF/MEK/ERK,^50^ we next measured the effect of CUDC‐907 on CXCL12‐mediated ERK signalling. We found that, while CXCL12 increased ERK phosphorylation, CUDC‐907 reduced the levels of P‐ERK in both stimulated and unstimulated cells (Figure 3E). Together, these data suggest that CUDC‐907 impairs CXCL12‐CXCR4 signals by decreasing sCXCR4 expression and phosphorylation levels, and thus blocks downstream signalling pathways.

### 3.5 CUDC‐907 enhances CLL cell sensitivity to other targeted inhibitors

**Figure 4:**
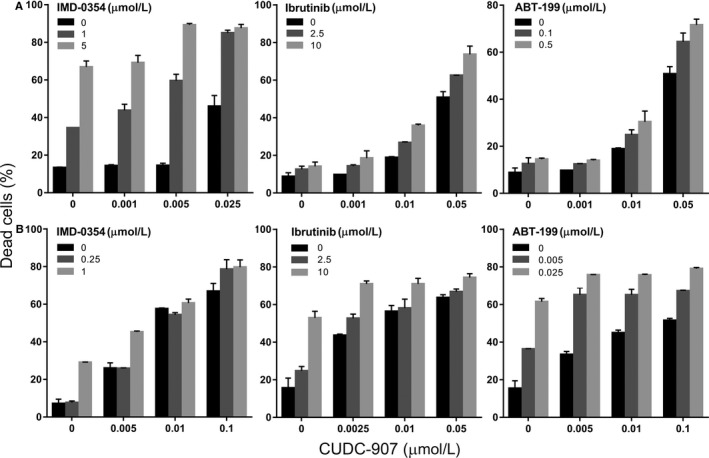
Combination of CUDC‐907 with other specific inhibitors show synergistic effects. A, Percentage of cell death, as measured by PI staining, in MEC‐1 cells treated with different concentrations of CUDC‐907 for 48 h, alone or in combination with IMD‐0354, ibrutinib or ABT‐199. Graphs show average of three independent experiments. B, Same in CLL patient cells cultured in the presence of 10 ng/mL IL‐4 and CD154 for 24 h before treated with drug combinations

Our results so far showed that CUDC‐907 is able to suppress different pro‐survival signalling pathways often activated in the microenvironment of proliferative centres, while effectively inducing apoptosis through different pathways. This lead to the hypothesis that CUDC‐907 could sensitize CLL cells to other drugs by interfering with their protective environment. Thus, we investigated the combination of CUDC‐907 with inhibitors that target other signalling pathways, such as those used in Figure 1. We found that the combination of CUDC‐907 with IMD‐0354, ibrutinib, or ABT‐199 increased MEC‐1 cell death (Figure 4A), to the point of being synergistic in most of the concentrations tested (Table S2). The enhancing effect of CUDC‐907 on IMD‐0354, ibrutinib, and ABT‐199 was also observed in CLL patient cells cultured in conditions that mimic the microenvironment of proliferation centres (Figure 4B). These combinations were also calculated to be synergistic (Table S2). Together, these results suggest that CUDC‐907 inhibits many signals involved in the resistance of CLL cells to treatment, which may help sensitize malignant B cells to other targeted treatments. This provides a rationale to further study the possibility of using CUDC‐907 as an adjuvant therapy in CLL.

## 4 DISCUSSION

There are many precision medicines currently being assessed clinically to treat B cell malignancies.^22^ However, CLL still presents therapeutic challenges, mostly due to the fact that malignant cells are resistant to many drugs when they reside in the bone marrow and lymphoid node microenvironments. It has been confirmed that these protective niches are critical for CLL cells escaping chemotherapy mediated apoptosis.^4^ It has also been shown that they are essential for CLL cell proliferation, survival, expansion, and clonal evolution.^7^ Thus, blocking the crosstalk of CLL cells with the microenvironment has been proposed to be a key strategy to induce apoptosis. In this study, we reported that CUDC‐907, a dual PI3K and HDAC inhibitor, has a strong impact on the microenvironment of malignant B cells and is highly effective in killing CLL cells.

The simultaneous inhibition of HDAC and PI3K signalling has been previously shown to promote the inhibition on tumour cell growth and induction of apoptosis in different cancers, including hematopoietic malignancies.^26^, ^27^ Indeed, CUDC‐907 has recently been shown to be safe and effective in patients with relapsed/refractory DLBCL.^30^ In hematopoietic cells, PI3K signalling activation is normally mediated by the p110δ isoform of PI3K, and it has been suggested that its inhibition has therapeutic value for CLL patients.^21^, ^51^, ^52^ CAL‐101 (idelalisib), a specific inhibitor of PI3Kδ, has shown a strong effect in the treatment of CLL cells in vitro and in vivo.^20^, ^21^ Recently, an inhibitor targeting both isoforms p110δ and p110γ (IPI‐145, duvelisib) showed even greater effects than idelalisib in the induction of apoptosis, inhibition of chemokine production, and migratory abilities of CLL cells.^53^ Thus, it is still debated whether isoform‐specific inhibition, instead of total PI3K inhibition, may be the optimal therapeutic strategy in lymphoid malignancies. In this study, CLL cells showed higher sensitivity to CUDC‐907 than idelalisib. This supports the hypothesis that pan‐PI3K inhibition may be more effective than blocking specific isoforms.

Our results show that malignant B cells are very sensitive to CUDC‐907, which induces cell death by engaging the intrinsic and extrinsic pathways of apoptosis. This is consistent with the fact that HDAC inhibition has previously been reported to induce cell death by various molecular mechanisms, activating both the extrinsic and intrinsic pathways.^23^ Apart from its direct apoptotic effects, our results demonstrate that CUDC‐907 may also have an impact on survival pathways normally activated in CLL cells. These provide essential proliferation signals for malignant B cells.^14^, ^15^, ^16^, ^17^, ^18^, ^19^ CUDC‐907 not only blocked the PI3K/AKT pathway but also simultaneously targeted the RAF/MEK/ERK, STAT3, and NF‐κB pathways. Moreover, it also inhibited the expression of anti‐apoptotic BCL‐2 family proteins such as BCL‐2, BCL‐xL, and MCL‐1.^39^, ^42^, ^43^, ^54^ Of note, the protective effects of AKT in CLL cells are largely dependent on the activation of the anti‐apoptotic protein MCL‐1.^55^ The specific mechanisms involved in these inhibitions are not clear and would require further investigations, but suggest that CUDC‐907 has other critical functions apart from the known inhibitory effects on HDACs and PI3K.

Indeed, CUDC‐907 had more effects on microenvironment‐related factors. It also decreased the expression of cytokines BAFF and APRIL and their receptors BAFFR, TACI, and BCMA. BAFF and APRIL induced the NF‐κB pathway, which is constitutively activated in stimulated CLL. NF‐κB activity normally correlates with chemoresistance and poorer clinical outcome.^14^, ^15^, ^16^ CUDC‐907 can inhibit NF‐κB signalling and thus reduce the protection that it exerts on CLL cells, which will decrease cell resistance and promote spontaneous or drug‐mediated apoptosis. Also, CUDC‐907 decreased the mRNA expression and secretion of CCL3 and 4, which are secreted by CLL cells in response to BCR signalling activation or culture with NLS cells and attract T cells to the microenvironment.^46^, ^47^, ^48^

CXCR4 is highly expressed on the surface of peripheral blood circulating CLL cells, and is important for CLL cell survival, migration, and interaction with the protective microenvironment.^10^, ^12^, ^56^, ^57^ CXCL12 ligands produced by stromal cells induce CLL cell chemotaxis and migration through activation of CXCR4.^11^, ^12^, ^13^ In this study, we found that CUDC‐907 not only reduced CXCL12‐induced smCXCR4 expression but also inhibited CXCR4 phosphorylation at Ser339. This could result in a rapid re‐distribution of CLL cells from spleens and lymph nodes into the circulation and prevent re‐entry into the microenvironment.^49^ This suggests a mechanism to further induce apoptosis in vivo, where CUDC‐907 may purge CLL cells from the protective lymph node and bone marrow niches.

Based on all these data, we propose that the pleiotropic effects of CUDC‐907 may be critical on its potent impact on CLL cell survival. Moreover, this reduction in pro‐survival pathways could help to sensitize CLL cells to other drugs by blocking protective signals. This hypothesis was supported by the fact that we observed a synergistic effect of CUDC‐907 with different concentrations of NF‐κB inhibitors (IMD‐0354), BTK inhibitor (ibrutinib), and BCL2 inhibitors (ABT‐199). Recently, we also reported that CUDC‐907 can be combined with MEK inhibitors to overcome the resistance of CLL cells to these drugs.^58^ The potential combinations of CUDC‐907 treatments could therefore be numerous. Importantly, the concentrations of CUDC‐907 needed to achieve the synergistic effects described here are in the nanomolar range, which have clinical relevance and would help reduce any off target effects of the drug. We conclude that the ability of CUDC‐907 to be combined at very low concentrations with other specific inhibitors to enhance their antitumour activity makes it an interesting candidate for clinical trials in B cell malignancies. Further in vivo experiments will be needed to evaluate this possibility.

## AUTHOR CONTRIBUTION

YC and SM designed the experiments, analysed the data and prepared the manuscript, with help from MJSD and SJ. YC performed the experiments, with help from CP, VS, SX, and GK‐F. BK, SW, and CB obtained the patient samples and provided clinical advice. All authors reviewed the manuscript.
