# Supplementary material for: CUDC‐907 blocks multiple pro‐survival signals and abrogates microenvironment protection in CLL
Source: J Cell Mol Med. 2018 Oct 24;23(1):340–8. doi: 10.1111/jcmm.13935 (PMC6307843; doi:10.1111/jcmm.13935)
Supplement: Supplementary file 1 [file JCMM-23-340-s001.pdf]

SUPPLEMENTARY INFORMATION

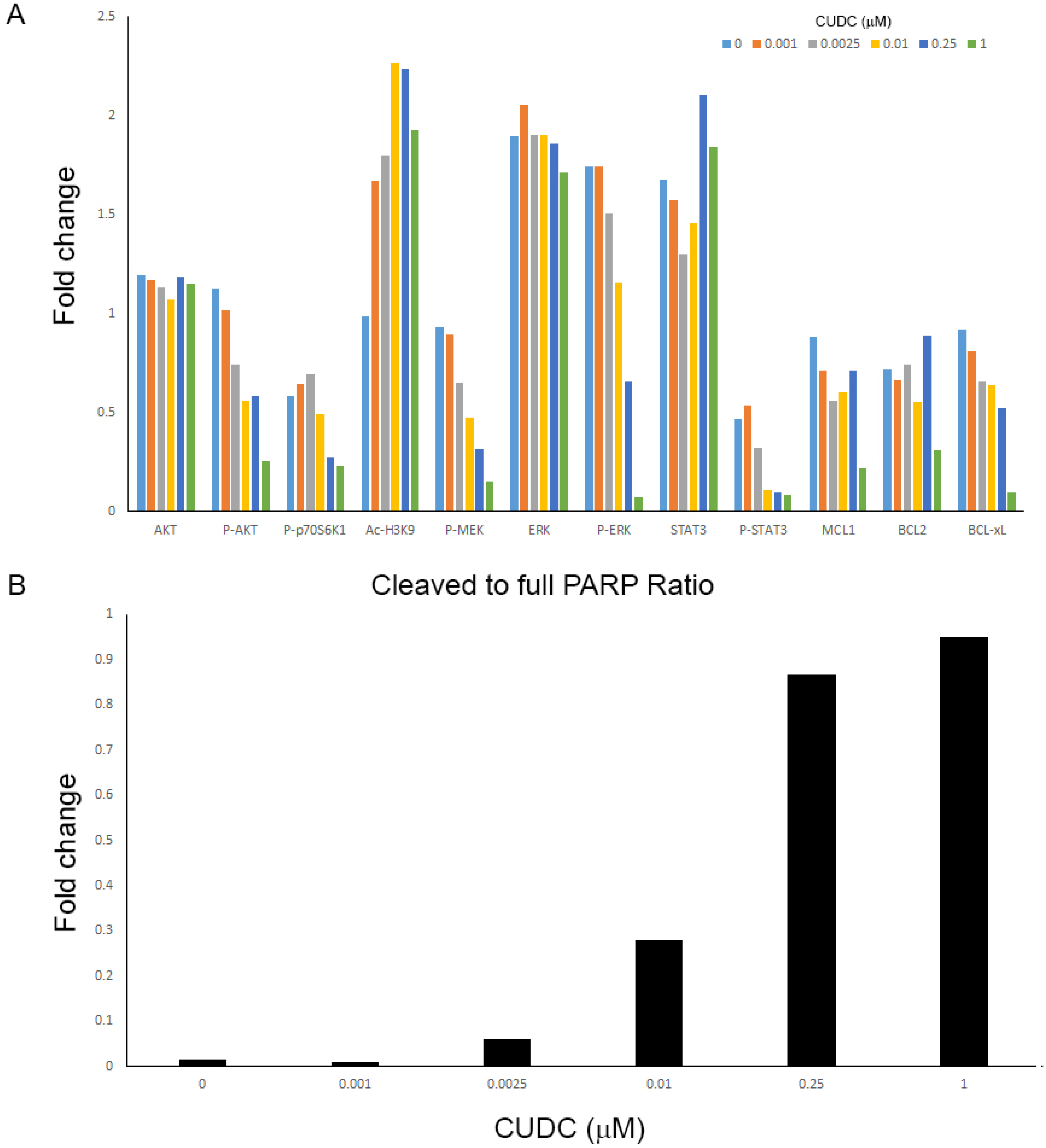

Figure S1. CUDC-907 inhibits pro-survival signals in CLL cells. Densitometry analysis of Figure 2A performed with Image J, showing values related to actin expression **(A)** or the ration between cleaved and non-cleaved PARP (**B**).

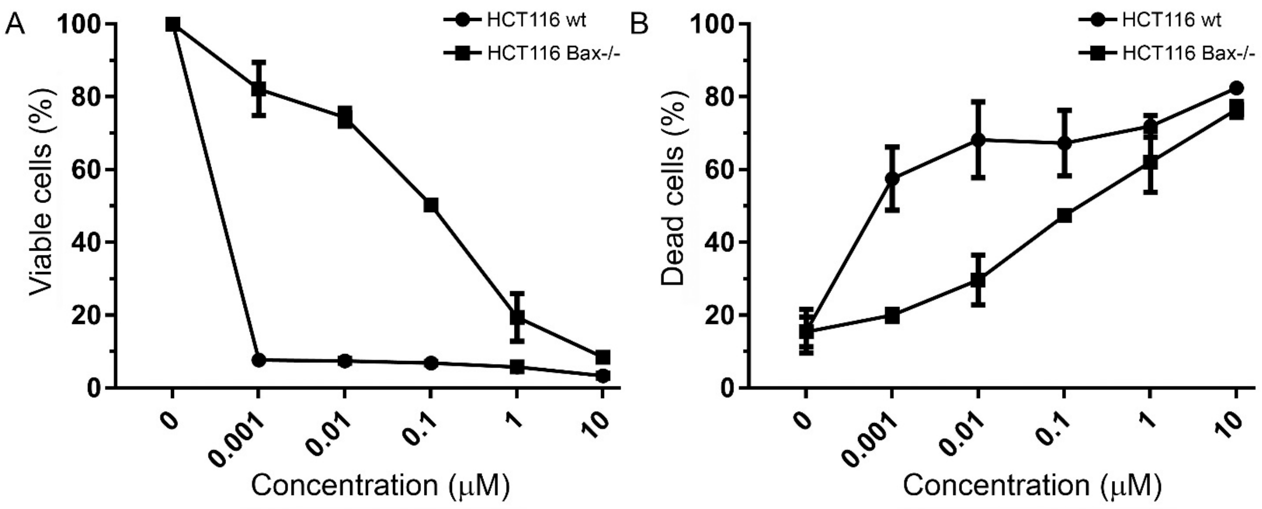

Figure S2. CUDC-907 induces cell death through Bax-dependent and -independent pathways. **(A)** HCT116 and HCT116 Bax$^{-/-}$ cell viability measured by MTS assay, normalized to Control samples (0μM). Cells were treated with different concentrations of CUDC-907 for 48 hours. All graphics show mean values of two independent experiments and error bars represent standard deviation. **(B)** Induction of cell death in the same cells, as measured by FACS analysis of Annexin V/PI-stained cells. Graph shows the percentage of double positive cells (apoptotic).

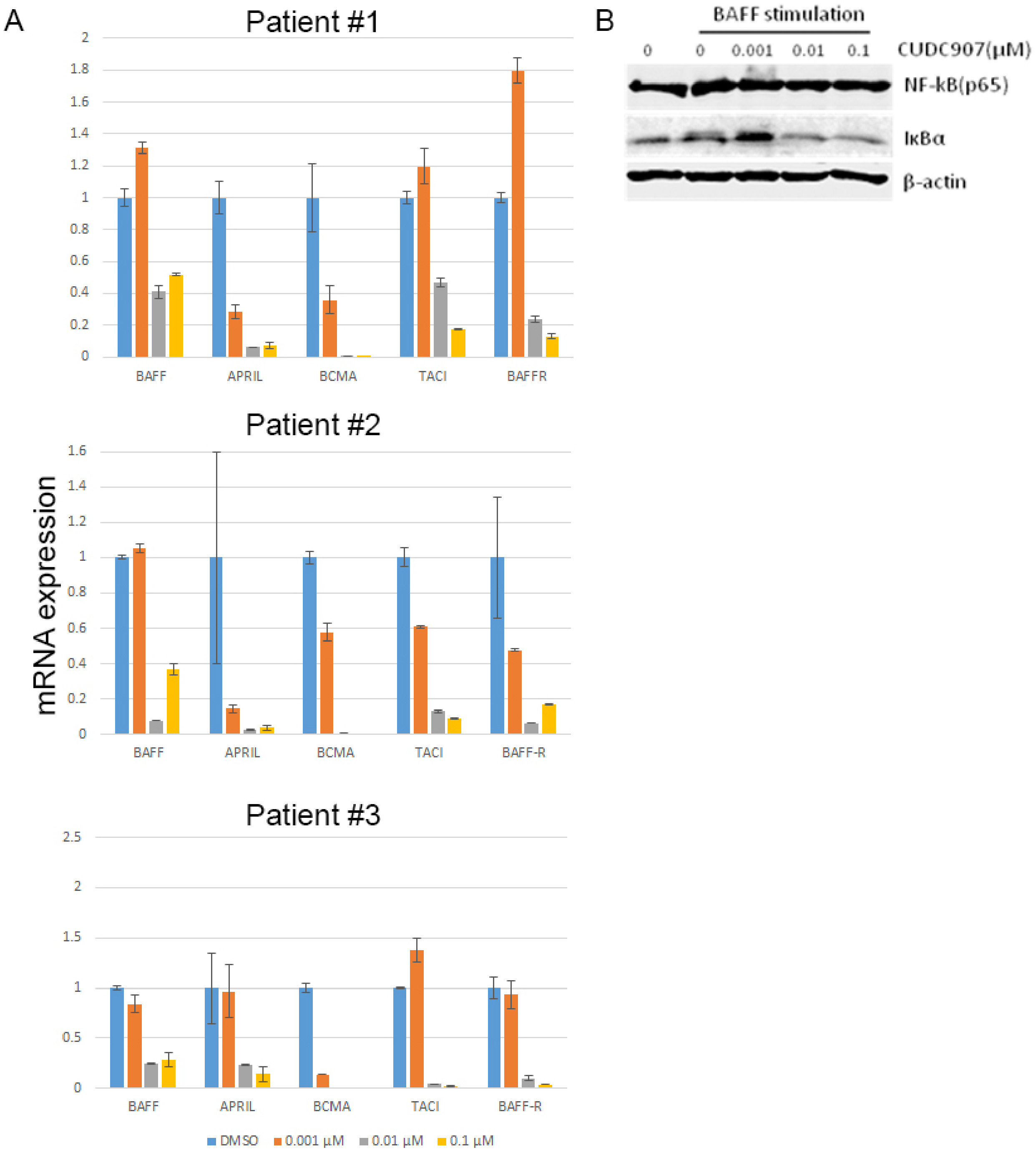

Figure S3. CUDC-907 induces cell death through Bax-dependent and -independent pathways. **(A)** Same as Figure 2C, using three more patient samples. **(B)** Same as Figure 2D, using another patient sample.

Gene	Forward primer (5'→3')	Reverse primer (5'→3')
BAFF	GGGAGCAGTCACGCCTTAC	GATCGGACAGAGGGGCTTT
APRIL	GTGTGGACGGGACAGTGAG	GCATCGGAACTCTGACAGTACAG
BCMA	ACCTTGTCAACTTCGATGTTCTT	CAGAGAATCGCATTCGTTCCTT
TACI	CAGGAGCCCAGTGAACCTTC	CCTTCCCGAGTTGTCTGAATTG
BAFFR	GGGTAGAGGCTCCGTACTCA	GGGCTTCATTTGACGGAGGA
CCL3	AGTTCTCTGCATCACTTGCTG	CGGCTTCGCTTGGTTAGGAA
CCL4	CTGTGCTGATCCCAGTGAATC	TCAGTTCAGTTCCAGGTCATACA
CCL17	CCAGGGATGCCATCGTTTTTG	AGTCAGGAGTCTGGGGTGAG
CCL22	ATCGCCTACAGACTGCACTC	GACGGTAACGGACGTAATCAC

Table S1. qPCR primers used in this study.

MEC-1

CUDC	IMD	CI	CUDC	Ibrutinib	CI	CUDC	ABT-199	CI
0.001	1	**0.80**	0.001	2.5	**0.56**	0.001	0.1	**1.80**
0.001	5	**0.67**	0.001	10	**0.24**	0.001	5	**1.51**
0.005	1	**0.31**	0.01	2.5	**0.86**	0.005	0.1	**0.88**
0.005	5	**0.08**	0.01	10	**0.40**	0.005	5	**0.58**
0.025	1	**0.04**	0.05	2.5	**0.29**	0.025	0.1	**0.59**
0.025	5	**0.11**	0.05	10	**0.11**	0.025	5	**0.24**
	AVERAGE	**0.33**		AVERAGE	**0.41**		AVERAGE	**0.93**

PATIENT CELLS

CUDC	IMD	CI	CUDC	Ibrutinib	CI	CUDC	ABT-199	CI
0.005	0.25	**2.83**	0.0025	2.5	**0.57**	0.005	0.005	**0.30**
0.005	1	**0.97**	0.0025	10	**0.43**	0.005	0.025	**0.51**
0.01	0.25	**0.49**	0.01	2.5	**0.75**	0.01	0.005	**0.31**
0.01	1	**0.55**	0.01	10	**0.49**	0.01	0.025	**0.51**
0.1	0.25	**0.42**	0.05	2.5	**0.83**	0.1	0.005	**0.33**
0.1	1	**0.47**	0.05	10	**0.53**	0.1	0.025	**0.42**
	AVERAGE	**0.95**		AVERAGE	**0.60**		AVERAGE	**0.40**

Table S2. Combination Index (CI) values calculated using the CalcuSyn software. CI is used as an indication of synergy in the combination treatments shown in Figure 4. Values represent: < 0.1, Very strong synergism; 0.1–0.3, Strong synergism; 0.3–0.7, Synergism; 0.7–0.85, Moderate synergism; 0.85–0.9, Slight synergism; 0.90–1.1, Nearly additive; 1.1–1.2, Slight antagonism; 1.2–1.45, Moderate antagonism; 1.45–3.3, Antagonism; 3.3–10, Strong antagonism;>10, Very strong antagonism. In green, synergistic combinations. In red, non-synergistic combinations. All concentrations are in µM. The results show average synergy in all combinations, with some of the concentrations reaching very strong or strong synergism and only three having a degree of antagonism.
